# Affinity Purification of NF1 Protein–Protein Interactors Identifies Keratins and Neurofibromin Itself as Binding Partners

**DOI:** 10.3390/genes10090650

**Published:** 2019-08-28

**Authors:** Rachel M. Carnes, Robert A. Kesterson, Bruce R. Korf, James A. Mobley, Deeann Wallis

**Affiliations:** 1 Department of Genetics, The University of Alabama at Birmingham, Birmingham, AL 35294, USA; 2 Department of Anesthesiology and Perioperative Medicine, The University of Alabama at Birmingham, Birmingham, AL 35294, USA

**Keywords:** neurofibromatosis Type I, neurofibromin, Ras, protein–protein interactors, binding partners, immunoprecipitation, affinity purification, mass spectrometry, keratins

## Abstract

Neurofibromatosis Type 1 (NF1) is caused by pathogenic variants in the *NF1* gene encoding neurofibromin. Definition of NF1 protein–protein interactions (PPIs) has been difficult and lacks replication, making it challenging to define binding partners that modulate its function. We created a novel tandem affinity purification (TAP) tag cloned in frame to the 3’ end of the full-length murine *Nf1* cDNA (*mNf1*). We show that this cDNA is functional and expresses neurofibromin, His-Tag, and can correct p-ERK/ERK ratios in *NF1* null HEK293 cells. We used this affinity tag to purify binding partners with Strep-Tactin^®^XT beads and subsequently, identified them via mass spectrometry (MS). We found the tagged mNf1 can affinity purify human neurofibromin and vice versa, indicating that neurofibromin oligomerizes. We identify 21 additional proteins with high confidence of interaction with neurofibromin. After Metacore network analysis of these 21 proteins, eight appear within the same network, primarily keratins regulated by estrogen receptors. Previously, we have shown that neurofibromin levels negatively regulate keratin expression. Here, we show through pharmacological inhibition that this is independent of Ras signaling, as the inhibitors, selumetinib and rapamycin, do not alter keratin expression. Further characterization of neurofibromin oligomerization and binding partners could aid in discovering new neurofibromin functions outside of Ras regulation, leading to novel drug targets.

## 1. Introduction

Neurofibromatosis Type 1 (NF1) occurs in ~1:3500 births due to pathogenic variants in the *NF1* gene that encodes the protein neurofibromin. NF1 is characterized primarily by benign tumors that form along nerves anywhere in the body, called neurofibromas. The NF1 phenotype is diverse and variable, even within the same family with the same mutation. Individuals with NF1 may also develop learning disabilities, macrocephaly, optic glioma, disfigurement, abnormalities of the bone, scoliosis, and hypertension; and are at an increased risk of developing malignant peripheral nerve sheath tumors (MPNSTs). Different cell types exhibit different phenotypes in NF1 patients. For example, melanocytes are involved in the café-au-lait macule (CALM) phenotype, while Schwann cells are associated with neurofibromas. 

*NF1* plays a significant role in cancer, as germline loss and homozygous inactivation lead to tumor formation in individuals with NF1. Further, somatic loss of *NF1* is common and found in many different types of cancers, including up to 87% of MPNST [1], 23% of acute lymphoblastic leukemia, 12%–18% of all melanomas, 11%–18% of glioblastoma, 12% of non-small-cell lung cancer, 12% of lung squamous-cell carcinoma, 13% of lung adenocarcinoma, 10%–14% of bladder urothelial carcinoma, 14% of uterine carcinosarcoma, 11%–12% of uterine endometrial carcinoma, 12% of ovarian serous cystadenocarcinoma, 11% of pancreatic carcinoma, 10% of metastatic cutaneous squamous-cell carcinoma, and 10% of gastric adenocarcinoma (reviewed by [2]). The identification of somatic *NF1* mutations in such a wide spectrum of tumors, including types not associated with NF1, indicates that neurofibromin is likely to play a key role in cancer beyond what is evident in the tumor predisposition syndrome NF1. Therapeutic approaches are necessary to address these phenotypes, but are not readily available due to limited understanding of neurofibromin regulation and additional functions, other than regulating Ras. 

As protein–protein interactions (PPIs) imply functional connections that may influence neurofibromin activity, identifying proteins with which neurofibromin interacts will increase our understanding of NF1. Several groups have reviewed neurofibromin protein structure and putative interacting partners [3,4,5]. These interacting partners have functions such as intracellular trafficking, neuronal differentiation, membrane localization, actin cytoskeleton remodeling, ubiquitylation, cell adhesion, and cell signaling. Unfortunately, a high-quality NF1 interactome has not been described. Further, binding partners may be cell-type-specific, adding to the complexity of the neurofibromin interactome. The Biological General Repository for Interaction Datasets (BioGRID) lists known PPIs and catalogs 118 unique neurofibromin interactions. Several of these PPIs were identified individually in a single study, and most studies used a different protein as “bait” to identify neurofibromin as “prey”. Outside of the three isoforms of Ras (HRas, KRas, and NRas), only three binding partners have been identified in more than one study: FAF2 [6,7], HTR6 [8,9], and SPRED1 [10,11]. 

FAF2 (aka ETEA/UBXD8) helps mediate ubiquitin-dependent degradation of misfolded endoplasmic reticulum proteins in endoplasmic reticulum-associated degradation (ERAD) [12]. In mammalian cells, FAF2 protein directly interacts with and negatively regulates neurofibromin by promoting its ubiquitin-dependent proteolysis. FAF2 interacts within the GRD domain [6]. Silencing of FAF2 expression increases neurofibromin levels and downregulates Ras activity [6]. NF1 is known to be regulated by proteolysis and Cul3, an E3 ubiquitin-protein ligase complex and a known FAF2 interacting partner [13,14,15].

HTR6 is a serotonin receptor whose activity is mediated by G proteins that stimulate adenylate cyclase. HTR6 is also an activating receptor of mTOR signaling [8]. When neurofibromin expression is suppressed, the HTR6 constitutive receptor activity is reduced too. Disrupting HTR6-neurofibromin interaction prevents agonist-independent HTR6-operated cAMP signaling in the prefrontal cortex, an effect that might underlie neuronal abnormalities in NF1 patients [9].

SPRED1 aids neurofibromin in inactivating Ras. SPRED1 directly binds neurofibromin within the GRD [10,11]. As SPRED1 localizes to the lipid raft/caveoli [16], it recruits neurofibromin to the membrane where NF1 can associate with Ras and inhibit ERK activation. A pathogenic *NF1* mutation (delM1215) within the SPRED1 binding site indicates that SPRED1-induced recruitment of NF1 to the membrane is necessary for proper function [11]. Although Ras-binding is not affected, this mutation alters SPRED1 binding and the ability to present neurofibromin to Ras at the right place and time. Hence, the final tertiary conformation or neurofibromin interaction with binding partners is crucial for the disease phenotype. Loss of function mutations in SPRED1 cause Legius syndrome and an inability of SPRED1 to downregulate the Ras-MAPK pathway [17]. Notably, Legius syndrome and NF1 have phenotypic overlap (including CALMs and freckling); however, no pathogenic *SPRED1* mutations have been reported in individuals with Lisch nodules, neurofibromas, or optic pathway gliomas (OPGs). 

Two other NF1 PPIs include 14-3-3β (YWHAB) and 14-3-3η (YWHAH). The interaction of 14-3-3 is mainly directed to the C-terminal domain (CTD) of neurofibromin, and the cAMP-dependent protein kinase (PKA)-dependent phosphorylation clustered on CTD-Ser (2576, 2578, 2580, 2813) and Thr (2556) is required for the interaction [18]. The increased phosphorylation and association of 14-3-3 negatively regulates the function of neurofibromin. These findings indicate that PKA phosphorylation followed by 14-3-3 protein interaction may modulate the biochemical and biological functions of neurofibromin [18]. In our proteomics study, we showed that 14-3-3σ is up-regulated when *NF1* is deleted in HEK293 cells and that restoration of neurofibromin expression leads to decreased/normalization of expression [19]. 14-3-3 proteins have also been implicated in other Rasopathy phenotypes, including cardio-facio-cutaneous syndrome [20] and Noonan syndrome [21]. 

Given the importance of NF1 PPIs, we developed and describe a novel tandem affinity purification tag linked to full-length murine *Nf1* cDNA (*mNf1*). This cDNA is expressed and is able to inhibit Ras signaling when transfected into *NF1* null HEK293 cells. We utilized this tagged NF1 cDNA to affinity purify binding partners. This led to identification of 21 proteins with a high confidence of truly interacting with neurofibromin. One of the binding partners we identified was neurofibromin itself, indicating that it oligomerizes. As this has several implications, we validated this interaction through reverse immunoprecipitation with a halo-tagged full-length human *NF1* cDNA (*hNF1*). In addition, we identified several keratins regulated by estrogen receptors as neurofibromin PPIs. This corroborates a prior study from our group in which we showed that neurofibromin can regulate levels of keratins. Herein, we demonstrate that this regulation occurs in a Ras-independent manner. Further characterization of neurofibromin oligomerization and putative PPIs will help define neurofibromin function and potential therapeutic targets. 

## 2. Materials and Methods

### 2.1. Tagged NF1 cDNAs and Empty Vector Controls

**Figure 1:**
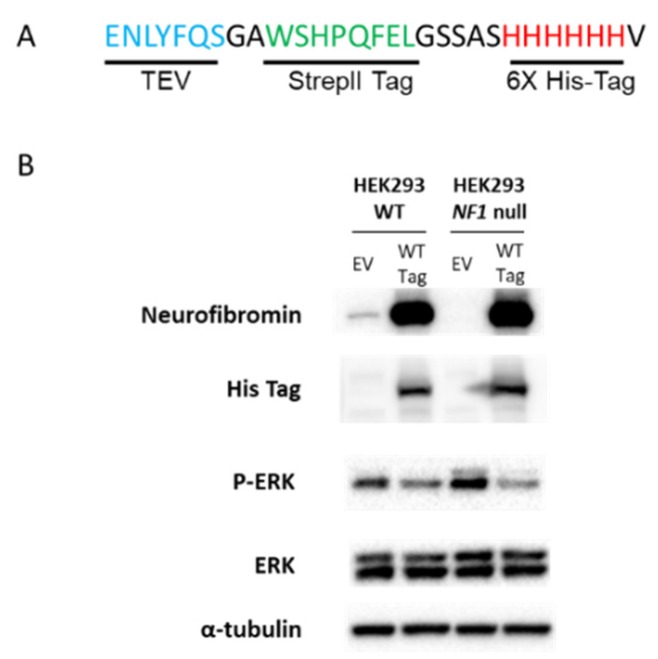
(**A**). Amino acid sequence of tandem affinity purification (TAP) tag cloned in frame with 3’ end of *mNf1* cDNA and empty vector control. Blue letters represent the TEV cleavage site. Green letters indicate StrepII tag. Red letters indicate 6XHis tag. (**B)**. Western blots showing neurofibromin protein levels in wildtype (WT, left) and *NF1* null HEK293 cells (right). Cells were transfected with either the Empty Vector (EV) or the tagged *mNf1* cDNA (WT) and probed with antibodies indicated. The first row of immunoblots shows the neurofibromin levels. The second row shows the functional His tag in only the lanes transfected with *mNf1* (WT). The third row shows pERK. Total ERK levels are shown in the fourth row. Fifth row shows α-Tubulin for normalization of NF1 levels.

We have developed and validated an *mNf1* cDNA expression system that allows us to examine the biochemical effects of any *NF1* genetic variant [22]. For this study, we added an affinity tag to the 3’ end of the *mNf1* cDNA. We used traditional subcloning to replace the BlpI and NotI restriction fragment in *mNf1* cDNA (GeneCopoeia NM_010897.2) with a similar fragment containing an in-frame insertion of ENLYFQSGAWSHPQFELGSSASHHHHHHVX amino acids between the penultimate valine and final stop codon (Figure 1A). These code for a TEV cleavage site, a StrepII tag, and a 6X His tag. We refer to this as “tagged” *mNf1* or WT. Tagged empty vector (EV) was created by inserting the same amino acid fragment in frame into pcDNA3.1 at KpnI and NotI sites. 

We have also obtained a heavily codon optimized 5’ Halo-tagged human *NF1 (hNF1)* cDNA along with an empty vector (EV) control (R777-E139 Hs. *NF1* was a gift from Dominic Esposito) (similar to Addgene plasmid #70,423; http://n2t.net/addgene:70423; RRID: Addgene_70423) that is stable in E. coli and non-toxic to human cells. The full-length cDNA sequences of endogenous *hNF1* and *mNf1* have 92% sequence identity with 0.08% mismatches; amino acid sequences share 98% identity [23]. This halo-tagged *hNF1* construct is so heavily codon optimized that when the sequence is used for a Blastn (nucleotide) search, the *NF1* gene sequence is not returned as a hit; yet a Blastp (protein) search reveals 100% identity to neurofibromin. GC content is increased from 43% in the endogenous *hNF1* to 60% in the codon optimized transcript. Codon usage is thought to affect transcription and translation efficiency, as well as mRNA stability and protein folding, thus potentially limiting the usefulness of this construct for some analyses.

### 2.2. Cell Lines and cDNA Transient Transfection

We used both HEK293 (WT) (obtained from ATCC) and *NF1* null HEK293 (null) cells in our studies. HEK293 *NF1* null cells were created via CRISPR-mediated targeting as described previously [22]. Cells were cultured in DMEM supplemented with 10% fetal bovine serum. We utilized HEK293 cells due to their historic use in NF1 research and particularly, in protein purification experiments, ease of transfection, and intact Ras signaling pathways. We previously reported the use of this cell line for multi-omics analysis [19] and to investigate *NF1* cDNA functions [22]. The tagged constructs were transfected into these cells following the LipoD293 DNA *in vitro* transfection protocol from SignaGen Laboratories (Rockville, MD, USA).

### 2.3. Affinity Purification (AP)

Strep Tag Affinity Purification: We utilized a StepTactin XT System from IBA Lifesciences (Gottingen, Gemany). Whole cell lysates were generated by collecting cells 36 h post-transfection and lysing with five freeze–thaw cycles in liquid nitrogen and a 37 °C water bath. A quantity of 10 μL of biotin blocking solution (iba Cat. #: 2-0205-050) was added to each sample before centrifuging the samples at 15,000 RPM for 45 min to clear lysates. Mag*Strep* “type3” XT beads (iba Cat. #: 2-4090-002) for *Strep*-tag^®^ purification were washed in 1X Buffer W (iba Cat. #: 2-1003-100). The supernatant from the cleared lysates was added to the tubes containing the beads and incubated on ice with vortexing every 10 min. After 30 min, the samples were placed on the magnetic separator (iba Cat. #: 2-1602-000). The supernatant was removed. The beads were washed three times with 1× Buffer W (iba Cat. #: 2-1003-100; Göttingen, Germany) before either being submitted to the UAB School of Medicine Mass Spectrometry/Proteomics Shared Facility or used for immediate Western blot analysis.

Halo Tag Affinity Purification: We used the Promega (Madison, WI, USA) HaloTag^®^ Mammalian Pull-Down System. Whole cell lysates were collected 36 h after transfection then centrifuged at 4000 RPM for 10 min at 4 °C. The supernatant was removed, and the cells were frozen at −80 °C for at least 30 min. Cells were lysed with Mammalian Lysis Buffer (Promega Cat. #: 6501). Protease inhibitor cocktail (Promega Cat. #: G6521) was added to the samples before centrifugation at 15,000 RPM for 30 min at 4 °C. The samples were diluted with 1X TBS then added to HaloLink™ Resin. The samples were incubated on an inversion table for 2 h at 4 °C. After incubation, the samples were centrifuged for 2 min at 3000 RPM and the supernatant was removed. The Halolink Resin was washed 4 times prior to TEV cleavage. TEV protease (Promega Cat. #: V109B) was incubated with the Halolink Resin for 1 h on ice with vortexing every 5 min, then centrifuged for 2 min at 3000 RPM. The eluate was placed into a new tube with Laemmli buffer and utilized for immediate Western blot analysis.

### 2.4. Reciprocal Immunoprecipitation

Both the Strep II/6X His tagged *mNf1* cDNA and Halo tagged *hNF1* cDNA were co-transfected into HEK293 *NF1* null cells. Then, either the Strep tag affinity purification or Halo tag affinity purification was carried out on the co-transfected cells. Once neurofibromin was purified with either of the protocols, the remaining protein was utilized for Western blot and denatured at 95 °C for 5 min. Samples were loaded on Bio-Rad (Hercules, CA, USA) 4%–20% gradient gels (Cat. #: 4568094) and run at 100 V for 2 h. The gels were transferred onto a polyvinylidene difluoride (PVDF) membrane at 100 V for 2 h. Blots were probed overnight at 4 °C with either the neurofibromin antibody (Cell Signaling Technologies Cat. #: 14623S; Danvers, MA, USA), the His tag antibody (GenScript Cat. #: A00186; Nanjing, China), or the Halo tag antibody (Promega Cat. #: G921A; Madison, WI, USA). The next morning, the blots were washed, and then probed with the respective secondary (Rabbit = Neurofibromin; Murine = His tag and Halo tag). They were washed three more times before imaging using chemiluminescent substrate from BioRad (Cat. #: 170-5061; Hercules, CA, USA) per the manufacturer’s protocols. 

### 2.5. Mass Spectrometry

Samples were eluted at 96 °C for 10 min in 1× LDS sample buffer. Eluate was collected in a fresh tube, reduced, denatured further at 70 °C for 10 min prior to loading onto a 10% Bis-tris gel. The gel was stained overnight with Colloidal Coomassie. Each lane was cut into eight molecular weight (MW) fractions. Each gel fraction was digested in trypsin overnight. Peptide digests (8 μL each) were injected onto a 1260 Infinity nHPLC stack (Agilent Technologies; Santa Clara, CA, USA), and separated using a 75 micron I.D. × 15 cm pulled tip C-18 column (Jupiter C-18 300 Å, 5 micron, Phenomenex). This system runs in-line with a Thermo Orbitrap Velos Pro hybrid mass spectrometer equipped with a nano-electrospray source (Thermo Fisher Scientific; Waltham, MA, USA), and all data were collected in CID mode. The nHPLC was configured with binary mobile phases that included solvent A (0.1%FA in ddH2O), and solvent B (0.1%FA in 15% ddH2O/85% ACN), programmed as follows; 10 min @ 5%B (3 μL/min, load), 90 min @ 5–40%B (linear: 0.5 nL/min, analyze), 5 min @ 70%B (3 μL/min, wash), 10 min @ 5%B (3 μL/min, equilibrate).

Following each parent ion scan (300–1200 m/z @ 60 k resolution), fragmentation data (MS2) was collected on the 15 most intense ions. For data-dependent scans, charge state screening and dynamic exclusion were enabled with a repeat count of 2, repeat duration of 30 s, and exclusion duration of 90 s. 

### 2.6. MS Data Conversion and Searches

The XCalibur RAW files were collected in profile mode, centroided and converted to MzXML using ReAdW v. 3.5.1. The mgf files were then created using MzXML2Search (included in TPP v. 3.5) for all scans. The data was searched using SEQUEST, which was set for two maximum missed cleavages, a precursor mass window of 20 ppm, trypsin digestion, variable modification C @ 57.0293, and M @ 15.9949. Searches were performed with a species-specific subset of the UniRef100 database. 

### 2.7. Peptide Filtering, Grouping, and Quantification

The list of peptide IDs generated based on SEQUEST (Thermo Fisher Scientific, Waltham, MA, USA) search results were filtered using Scaffold (Protein Sciences, Portland Oregon, OR, USA). Scaffold filters and groups all peptides to generate and retain only high-confidence IDs, while also generating normalized spectral counts (N-SC’s) across all samples for the purpose of relative quantification. The filter cut-off values were set with minimum peptide length of >5 AA’s, with no MH+1 charge states, with peptide probabilities of >80% C.I., and with the number of peptides per protein ≥2. The protein probabilities were then set to a >99.0% C.I., and a false discovery rate (FDR) <1.0. Scaffold incorporates the two most common methods for statistical validation of large proteome datasets, the false discovery rate FDR and protein probability [24,25,26]. Relative quantification across experiments were then performed via spectral counting [27,28], and when relevant, spectral count abundances were normalized between samples [29]. From these analyses, we identified over 800 unique peptides in our samples. 

### 2.8. Statistical Analysis

We performed 4 replicates of each affinity purification using wildtype tagged *mNf1* cDNA (WT) and tagged empty vector control (EV) in *NF1* null HEK293 cells. EV controls were utilized to filter out non-specific PPIs. All lists from each individual pull down were compiled (Supplementary Table S1). From this combined list of over 800 unique proteins, we filtered by comparing EV and WT protein lists. For the affinity purification data generated, two separate non-parametric statistical analyses were performed between each pair-wise comparison. These non-parametric analyses include 1) the calculation of weight values by significance analysis of microarray (SAM; cut off >|0.6|combined with 2) T-Test (single tail, unequal variance, cut off of *p* < 0.1), which then were sorted according to the highest statistical relevance in each comparison. For SAM, whereby the weight value (W) is a statistically derived function that approaches significance as the distance between the means (μ1 − μ2) for each group increases, and the SD (δ1 − δ2) decreases using the formula, W = (μ1 − μ2)/(δ1 − δ2). For protein abundance ratios determined with N-SC’s, we set a 2-fold change as the threshold for significance. In each case, all of the three tests (SAM, *t*-test, or fold change) had to pass as detailed above for final inclusion. Alternatively, if they were pulled down with WT *mNf1* but not EV, and had a spectral count of at least 4; these were also identified as hits. These were combined to create a finalized high-confidence list of neurofibromin interactors. 

### 2.9. Systems Analysis

Network analysis was carried out using MetaCore (GeneGO Inc., St. Joseph, MI, USA). Interactions identified within MetaCore are manually correlated using full-text articles. Detailed algorithms have been described previously [30,31].

## 3. Results

### 3.1. Neurofibromin Oligomerization

We cloned a tandem affinity tag in frame with the 3’ end of the murine *Nf1* cDNA. The amino acid sequence of this tag (depicted in Figure 1A) is composed of a TEV cleavage site, a StrepII tag, and a 6X His tag. We show that the tagged *mNf1* cDNA abundantly expresses neurofibromin in HEK293 cells (Figure 1B, row 1). Detection via anti-His indicates that the tag is also expressed (Figure 1B, row 2). Further, expression of tagged *mNf1* is able to repress pERK/ERK ratios in *NF1* null HEK293 cells (Figure 1B rows 3,4), which is consistent with our previous data with an untagged version of the *mNf1* cDNA [22]. This indicates tagged mNf1 retains GRD function.

**Figure 2:**
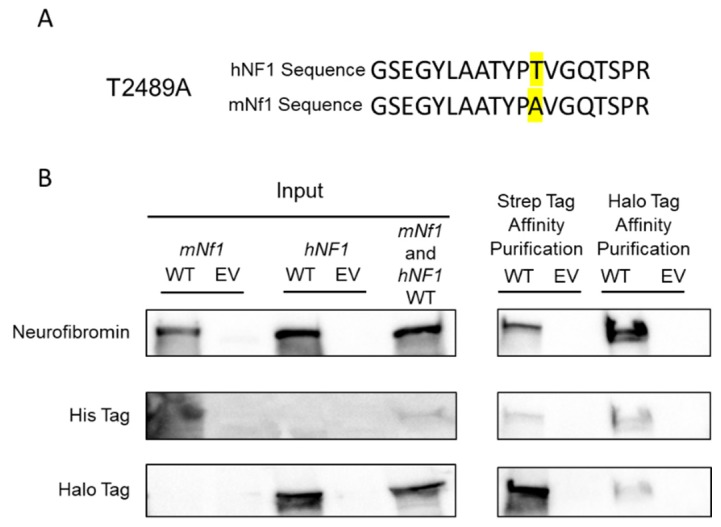
(**A**). The top row details the amino acid sequence of the fragment of human NF1 protein that affinity purified from WT HEK293 cells with the tagged *mNf1* cDNA. The second row depicts the homologous mouse neurofibromin protein sequence and highlights the non-conserved amino acid residue T2489A. (**B**). Reciprocal immunoprecipitation of the tagged murine and human cDNAs. The left-hand side shows blots of input lysates after cell transfections with each indicated cDNA prior to affinity purification. The right-hand side shows blots after affinity purification. The left two columns show the Strep tag affinity purification and the right two columns show the Halo tag affinity purification. WT indicates the co-transfection of both cDNAs into HEK293 *NF1* null cells; while EV indicates the empty vector transfection into the same cells. The first row of immunoblots is the visualization of neurofibromin. The second row shows the His tag from the *mNf1* cDNA. The third row depicts the Halo tag from the *hNF1* cDNA. The His tag is visible in the Halo immunoprecipitation WT lane and the Halo tag is visible in the Strep tag affinity purification WT lane, indicating that the two tagged neurofibromins are purifying the reciprocal neurofibromin when the individual affinity purification is completed.

Our initial experiments utilized the *mNf1* tagged cDNA in both *NF1*-replete or wildtype (WT) HEK293 cells and *NF1*-deficient (null) HEK293 cells. We noted that when we utilized HEK293 WT cells, we were able to affinity purify an endogenous human neurofibromin protein fragment with the tagged *mNf1* cDNA. The human NF1 protein fragment identified by MS is depicted in Figure 2A aligned with the mouse sequence. We were able to make this species determination based on one of the few amino acid changes between the human and murine protein sequences. Note, that the human T2489 is encoded by alanine in mouse. Due to the high homology between human and mouse neurofibromin proteins, we were only able to identify one fragment with sequence variation. This data suggests that neurofibromin may oligomerize with other neurofibromin proteins within the cell. In order to show that neurofibromin oligomerizes, we co-transfected a halo-tagged human *NF1* cDNA along with the tagged *mNf1* cDNA into the HEK293 *NF1* null cells, and then affinity purified with either the Strep tag or Halo tag. We confirmed that neurofibromin oligomerizes via these reciprocal immunoprecipitations (Figure 2B). We are able to detect halo-tagged hNF1 protein after AP with Strep tag and His-tagged mNf1 after AP with halo-tagged hNF1. 

### 3.2. Neurofibromin PPIs

AP-MS offers an unbiased approach for identifying PPIs and identifying protein complexes instead of binary interactions. Overexpression of a tagged protein is commonly used. Our affinity purifications using the tagged *mNf1* cDNA led to the identification of over 800 proteins (raw unfiltered data is provided in Supplementary Table S1). By comparing results from tagged neurofibromin and tagged empty vector we are easily able to distinguish bona fide interacting proteins from background noise. After more stringent analyses, a total of 21 proteins interacting with neurofibromin with high confidence were identified (Table 1). 

**Figure 3:**
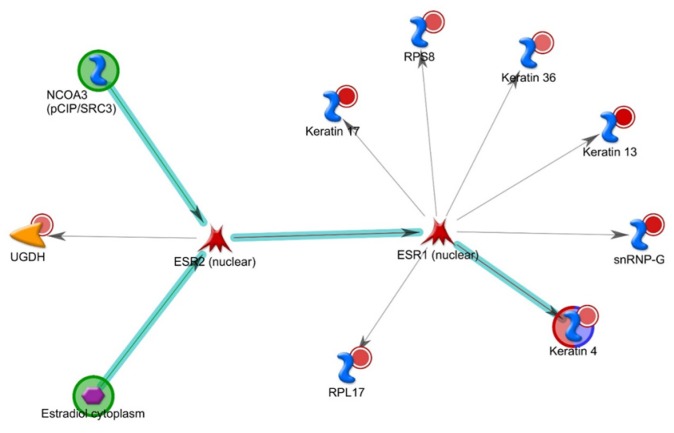
Metacore network analysis identifies eight of the top 21 interactors within the same estrogen receptor protein network.

When these 21 PPIs are utilized for Metacore network analysis, the topmost significant network is depicted in Figure 3 (*p* < 2.15 × 10^−29^). This estrogen receptor (ESR) network illustrates a pathway including eight of the proteins we identified (RPS8, RPL17, Keratins 4, 13, 17, and 36/Ha6, snRNP-G and UGDH) and three proteins that we did not identify (ESR2 (nuclear), ESR1 (nuclear) and NCOA3). Clearly, many of the keratins that we have identified (4, 13, 17, and 36/Ha6) via affinity purification are driving this process. 

### 3.3. Neurofibromin and Keratins

**Figure 4:**
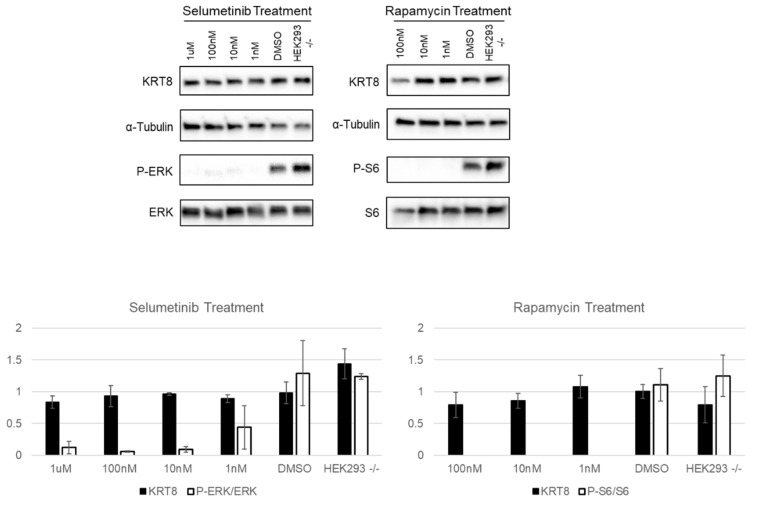
Keratin expression is unaffected by inhibition of Ras signaling pathways. NF1 null HEK293 cells were treated in dose response indicated with selumetinib or rapamycin and KRT8 levels (first row of bands) were evaluated by Western blot and normalized by α-tubulin (second row of bands). p-ERK/ERK ratios were also evaluated in response to selumetinib and pS6/S6 ratios in response to rapamycin to show drug efficacy (third and fourth rows of bands). Blots show an individual representative experiment. There is no statistical difference in KRT8 levels after *N* ≥ 3 represented in the histogram below the blots. p-ERK/ERK and pS6/S6 respond positively to selumetinib and rapamycin, respectively. Black bars indicate KRT8/tubulin ratios after treatment and white bars represent p-ERK/ERK or pS6/S6 ratios after treatment. (Rapamycin is toxic at doses higher than 100 nM). Error bars represent SEM.

Our affinity purifications indicate that neurofibromin interacts with several keratins. Our own multi-omics data indicate that keratins, including KRT8, are over expressed in *NF1* null cells by 8-fold over WT cells (both HEK293 and iPSCs); we further showed that Keratin expression was dependent on neurofibromin expression [19]. When NF1 was re-expressed in *NF1* null HEK293 cells, KRT8 levels decreased. Hence, NF1 directly binds keratins and can inhibit their expression. This inhibition appears to be Ras-independent; as when we block Ras signaling with pharmacological inhibitors including selumetinib (an MEK inhibitor) and rapamycin (an mTOR inhibitor) KRT8 expression does not change (Figure 4). p-ERK/ERK ratios were also evaluated in response to selumetinib and pS6/S6 ratios in response to rapamycin to show drug efficacy; both ratios dramatically decrease with treatment indicating that the drugs are effectively blocking Ras signaling but not altering KRT8 expression (Figure 4). This suggests that NF1 has functions outside of Ras inhibition. These functions may include keratin regulation and its downstream functions such as invasion and cell proliferation. 

### 3.4. Correlation with the Cancer Genome Atlas (TCGA)

Because proteins encoded by mutated genes in inherited genetic disorders are likely to interact with proteins known to cause similar disorders, suggesting the existence of disease PPI subnetworks, we further analyzed the correlations between cancer cases and NF1 PPIs. We evaluated the total number of cases associated with each of our PPIs in TCGA and intersected this with the number of cases associated with *NF1* (a total of 1564 cases). The data for each PPI are listed in the third column of Table 1 and graphically depicted in Supplementary Figure S1. For example, Keratin 17 is associated with 862 TCGA cases. Of these, 471 are also associated with *NF1* cases (471/862 = 54.6%). All of these PPIs have cases that are also associated with *NF1* at rates between 20%–55%. The keratins especially seem to have the highest rates with K13, 17, 31/Ha1, and 36/Ha6 all associating with *NF1* cases approximately 55% of the time. This is a very strong indication that these protein functions are coordinated to result in these phenotypes. 

## 4. Discussion

Using a novel cDNA expression system and an affinity tag, we were able to identify many novel neurofibromin binding partners, including neurofibromin itself. Mass-spectrometry-based studies are notorious for returning off-target and/or false positives. Hence, our data need to be followed up with independent experimental studies to verify interactions. For example, recent data from the Esposito lab also indicates neurofibromin dimerizes with itself (Dominic Esposito, personal communication). In addition, it has been reported that when synthetic NF1 is produced recombinantly in insect cells and purified, the purified protein is biochemically active and elutes as a putative dimer in size exclusion chromatography [32]. Unfortunately, no data is shown. Oligomerization/dimerization of neurofibromin may have interesting implications and may explain some unusual clinical findings. For example, if neurofibromin oligomerizes and one allele is mutated, this may “poison” the oligomer and result in dominant negative functions. We have previously noted that some *Nf1* cDNA mutations such as R681X lead to even more hyperactive Ras signaling and increased levels of GTP-Ras when compared to no *Nf1* cDNA (i.e., empty vector (EV) controls) [22]. In addition, a more severe phenotype for this mutation was also noted in mouse models with this mutation in comparison to null mutations [33]. Furthermore, a recent paper describes breast cancer in NF1 and shows a lack of large or whole gene deletions and an excess of nonsense and missense mutations [34]. These breast cancer mutation data are consistent with a dominant negative model of neurofibromin activity wherein some mutations are conferring risk of breast cancer due to mutant neurofibromin proteins disrupting normal neurofibromin oligomeric complexes. Hence, this may have clinical implications for genotype–phenotype correlations, as well as therapeutic implications. 

A few neurofibromin PPIs have already been associated with NF1: B-arrestin-2 and SF3B1. B-arrestin-2 regulates agonist-mediated G-protein coupled receptor signaling. Peripheral nerve Schwann cells (SC) show tonic ATP-mediated inhibition of cell proliferation, which requires P2Y2 and arrestins [35]. ATP-mediated growth suppression was reduced in mouse and human SCs with inactivating mutations in the *NF1* gene. *Nf1* mutant cells showed failure of arrestin-mediated suppression of GPCR-mediated Ca2+ release and of arrestin-mediated, PP2A-dependent, de-phosphorylation of Akt. Coover et al. suggest that the most direct explanation of these results is that arrestin function is modulated, directly or indirectly, by neurofibromin. Our data indicates that neurofibromin binds B-arrestin-2, suggesting this may be a direct modulation. Neurofibromin-regulated GPCR-arrestin signaling may contribute to the aberrant activity of a broad array of receptors, and thus, a number of clinical manifestations [36]. Hence, our data supports the conclusion that restoration of purinergic signaling to effect growth suppression may augment current approaches for therapy in *NF1* mutant nerve tumors [35]. Both neurofibromin and SF3B1 have been identified as oncogenic drivers in a variety of cancers. Different oncogenic drivers may be found in various subtypes of melanoma with neurofibromin prominent in acral melanoma and SF3B1 prominent in mucosal melanoma [37]. In fact, alterations to driver genes such as *SF3B1* and *NF1* are relatively common in mucosal melanoma [38]. Oncogenic driver events are also found in the majority (93%) of anorectal melanomas with *NF1* in 20% of cases and *SF3B1* mutations as a recurrent genetic event in mucosal melanomas [39]. *NF1* and *SF3B1* are among several genes in a minimum recommended list to provide relevant clinical information for the management of most Chronic Myeloid Neoplasms [40]. Myelodysplastic syndromes are shaped by gene aberrations involved in *NF1* and *SF3B1* [41,42,43]. Hence, though seemingly disparate, as SF3B1 is characterized as an RNA-splicing protein and neurofibromin as a Ras pathway protein, our finding that these proteins physically interact provides further evidence that they play a role in a common pathway leading to these types of cancers. 

Keratins are expressed in pairwise, tissue-specific, and developmentally dependent manners. Besides being well-known and characterized structural proteins important for protection from mechanical stress, the role of keratins in cell proliferation, differentiation, and transformation are beginning to be recognized. Keratins are widely used to identify epithelia, to characterize normal and transformed epithelia, to mark invasive and noninvasive tumor margins, and to predict survival of cancer patients. Use of keratins as diagnostic markers in tumor pathology is their most common application in cancer. Altered keratin expression during and after malignant transformation is reported to modulate signaling pathways involved in tumor progression [44]. For example, Keratin 17 is rapidly induced in wounded stratified epithelia and regulates cell growth by binding to 14-3-3σ and stimulating the Akt/mTOR pathway [45]. KRT17 can enhance the proliferation, migration, and invasion capacities of lung adenocarcinoma cells, thereby promoting tumor progression [46]. When Keratin 8 is ectopically expressed in mice, epidermis showed severe epidermal and hair follicle dysplasia with concomitant alteration in epidermal differentiation markers that progressively develop into premalignant areas in the aging animals [47]. Ectopic KRT8 also results in abnormal epidermal differentiation and a dramatic increase in the malignant progression of the skin tumors. 

It has been shown that neurofibromin co-localizes with keratin during early development of the human epidermis [48]. During this early fetal period, neurofibromin is abundantly expressed in epidermis and associated with cytokeratin filaments. This period is characterized by the initiation of differentiation of the basal cells, maturation of the basement membrane zone, and accentuated formation of selected cellular junctions. Hence, neurofibromin may function in the regulation of epidermal histogenesis by controlling the organization of the keratin cytoskeleton during the assembly of desmosomes and hemidesmosomes. Another study looking at neurofibromin binding partners also identified numerous different keratins including 4, 13, 17, but these were subsequently filtered out as potentially non-specific [49]. 

Our network analysis identified a single network that includes eight of our PPIs. Though we did not identify ESR2, a central protein in this estrogen receptor network (Figure 3), it has been shown to associate with neurofibromin in breast cancer cells by AP-MS with tagged ESR2 [50]. Furthermore, a role for hormonally induced NF1 pathophysiology has long been suspected. Clinical features such as cutaneous neurofibromas begin to develop around puberty, and the number and size of neurofibromas increase during pregnancy [51]. *Nf1*-deficient rat models were recently developed to evaluate the effect of *Nf1* on tumorigenesis; *Nf1* mammary tumors are positive for estrogen receptor and highly express pan-cytokeratins [52]. K18 may play a regulatory role in hormonally responsive breast cancer, as it can associate with and sequester the ERα target gene and ERα coactivator LRP16 in the cytoplasm, thus attenuating ERα-mediated signaling and estrogen-stimulated cell cycle progression in breast tumor cells [53]. Hence, this network indicates a new role for neurofibromin interacting with estrogen receptors and keratins to affect tumorigenesis. This function for neurofibromin may be independent of Ras. This provides a novel therapeutic target for tumors that utilize this mechanism. 

Since neurofibromin was discovered, there have been several attempts to identify binding partners. Of the 37 studies included in BioGrid, only 12 focus directly on neurofibromin binding partners [10,11,18,54,55,56,57,58,59,60,61,62]. Most studies only highlight one or two significant PPIs. The primary exception is the Li et al. study, which includes a comprehensive list of 49 binding partners discovered via affinity purification using antibodies against endogenous NF1 proteins followed by mass spectrometry analysis (AP-MS) [49]. This study highlights proteins that are involved in mTOR signaling, namely LAMTOR1 (Late Endosomal/Lysosomal Adaptor, MAPK And MTOR Activator 1). Our study is unique among others because we are the first to have a full-length tagged *mNf1* cDNA. This gives us unparalleled sensitivity to begin cataloging neurofibromin binding partners in various cell types. 

There has been little replication of neurofibromin PPIs among studies. Within BioGrid, there are only three proteins that have overlap between studies: FAF2, SPRED1, and HTR6. Though the studies that identified these three proteins mostly utilized HEK293T or HEK293 cells, there is a difference between how the proteins were purified. Only one of the studies that identified FAF2 and neurofibromin did the reverse IP to show that neurofibromin was also purifying FAF2 [6]. Phan et al., focused mainly on the yeast ortholog of neurofibromin, Ira2 [6]. When utilizing human neurofibromin for reverse IP, they cut the protein into sections before adding a Flag tag to each section to use as bait for FAF2. The same can be said about the two studies that identified SPRED1 as a binding partner of neurofibromin. One only looked at the interaction in terms of SPRED1 [11], while the other used short sections of Flag-tagged neurofibromin to understand the interaction between SPRED1 and neurofibromin [10]. Similarly, one study of HTR6 only focused on HTR6 [8] and the other evaluates both [9]. The latter study utilized mouse striatum tissue to show that neurofibromin binds HTR6. 

Protein abundance and cell-type specificity also play roles in identification of PPIs. Despite the fact that neurofibromin is well-known to bind Ras, until recently, no study had been able to detect this interaction [4,49]. This may be because of the transient nature of the interaction or the Ras abundance in specific cell types. KRas was detected in HeLa cells where it is more abundantly expressed, but not in HEK293T cells where it has relatively low expression [49]. Hence, neurofibromin interacting partners are cell-type specific. Indeed, in this same study, only 51% of neurofibromin PPIs identified in HeLa cells overlap with those identified in HEK293T cells. As many of the studies in BioGrid use different cell types, eighteen percent of the previously reported NF1-interacting proteins were not detectable in at least one of the two cell lines used, and 28% of the previously reported proteins passed specificity filtration criteria in only one cell line [49]. These results indicate that cell line differences, especially protein abundance differences, will affect the formation of cell-specific protein interactions. We will, therefore, need to study neurofibromin binding partners in specific, clinically relevant cell types to better understand its functions and pleomorphic phenotype. 

Experimental methods including subcellular enrichment, tags, antibodies, and lysis buffers may also help determine which proteins are retrieved. Different tags can affect the folding of the protein or fold the protein in a way that interferes with certain binding partners. Studies that employed a neurofibromin antibody for immunoprecipitation will contain non-specific binding partners as the current neurofibromin antibodies react to non-specific proteins. 

In summary, we have created a tagged *mNf1* cDNA that can be expressed in human cells and retain function. We have used it to identify novel neurofibromin PPIs. Our data suggest that neurofibromin oligomerizes, which may have implications for genotype–phenotype correlations and therapeutic choices. Furthermore, we identify a network involving estrogen receptors and keratins that may contribute to neurofibromin’s role in oncogenesis. 

## Figures and Tables

**Table 1:** High-Confidence NF1 protein–protein interactions (PPIs).

UniProtKB ID	UniProt	Function	TCGA Overlap with NF1/Total Cases
40S ribosomal protein S8	P62241	Ribosomal protein that is a part of the 40S subunit. Increased gene expression seen in colorectal tumors and colon polyps.	177/755
60S ribosomal protein L17	P18621	Ribosomal protein that is a part of the L22P family and is a component of the 60S subunit.	136/527
βETA-arrestin-2	P32121	Regulates agonist-mediated G-protein coupled receptor (GPCR) signaling through desensitization and resensitization.	189/593
Dermcidin	P81605	The N-terminal peptide promotes neural cell survival under oxidative stress. Has oncogenic effects in breast tumors.	141/579
Eukaryotic translation initiation factor 3 subunit K	Q9UBQ5	Component of the eIF3 complex which associates with the 40S ribosome to help initiate protein synthesis.	248/954
Insulin-like growth factor 2 mRNA-binding protein 2	Q9Y6M1	RNA-binding factor that recruits target transcripts to cytoplasmic protein-RNA complexes (mRNPs).	440/1729
Interferon-induced protein with tetratricopeptide repeats 3	O14879	An antiviral protein which acts as an inhibitor of cellular and viral processes, such as cell migration, proliferation, signaling, and viral replication.	192/965
Keratin 13	A1A4E9	Member of the type I cytokeratin family. Works in tandem with keratin 4	465/846
Keratin, type I cuticular Ha1	Q15323	Member of the type I cytokeratin family.	
Keratin, type I cuticular Ha6	O76013	Member of the type I cytokeratin family.	458/825
Keratin, type I cytoskeletal 17	Q04695	Member of the type I cytokeratin family involved in determing the shape and direction of hair development.	471/862
Keratin, type II cytoskeletal 4	P19013	Member of the type II cytokeratin family. Heterodimerizes with keratin 13.	188/757
Large proline-rich protein BAG6	P46379	An ATP-independent molecular chaperone preventing the aggregation of misfolded and hydrophobic patches-containing proteins	238/933
Low-density lipoprotein receptor-related protein 2	P98164	Acts together with CUBN to mediate endocytosis of high-density lipoproteins	395/1525
Nuclear protein localization protein 4 homolog	Q8TAT6	Forms a ternary complex with UFD1 and VCP which transports misfolded proteins from the ER to the cytoplasm for proteasomal degradation.	425/1399
Proteasome inhibitor PI31 subunit	Q92530	Inhibits the hydrolysis of peptides by the 20S proteasome	208/804
Protein disulfide-isomerase A4	P13667	Catalyzes the rearrangement of disulfide bonds within proteins.	274/1211
Regulator of G-protein signaling 10	O43665	Regulates GPCR signaling cascades by driving the G-protein α subunit into its GDP bound form.	201/854
Small nuclear ribonucleoprotein G	P62308	Major component of the SMN-Sm complex which takes part in splicing pre-mRNAs.	107/446
Splicing factor 3B subunit 1	O75533	Component of the SF3B complex which takes part in splicing pre-mRNAs.	249/958
UDP-glucose 6-dehydrogenase	O60701	Catalyzes the formation of UDP-α-D-glucuronate. Required for embryonic development via its role in the biosynthesis of glycosaminoglycans.	143/481

## Supplementary Materials

The following are available online at https://www.mdpi.com/2073-4425/10/9/650/s1, Table S1: Total spectrum count.
